# Invasive rat control is an efficient, yet insufficient, method for recovery of the critically endangered Hawaiian plant hau kuahiwi (*Hibiscadelphus giffardianus*)

**DOI:** 10.1371/journal.pone.0208106

**Published:** 2018-11-28

**Authors:** Nathan S. Gill, Stephanie Yelenik, Paul Banko, Christopher B. Dixon, Kelly Jaenecke, Robert Peck

**Affiliations:** 1 U.S. Geological Survey, Pacific Island Ecosystems Research Center, Hawai’i Volcanoes National Park, HI, United States of America; 2 Department of Integrative Biology, University of Wisconsin-Madison, Madison, WI, United States of America; 3 Department of Statistics, Brigham Young University, Provo, UT, United States of America; 4 Hawai’i Cooperative Studies Unit, University of Hawai’i at Hilo, Hawai’i Volcanoes National Park, HI, United States of America; East Carolina University, UNITED STATES

## Abstract

Biological invasions of rodents and other species have been especially problematic on tropical islands. Invasive *Rattus rattus* consumption of *Hibiscadelphus giffardianus* (Malvaceae; common Hawaiian name hau kuahiwi) fruit and seeds has been hypothesized to be the most-limiting factor inhibiting the critically endangered tree, but this has not been experimentally tested, and little is known about other factors affecting seed dispersal, germination, and seedling establishment. Thus, we do not know if rat removal is sufficient to increase hau kuahiwi recruitment. This study aims to evaluate the effect of rat population control on the ability of hau kuahiwi to retain fruit and establish seedlings. We compared hau kuahiwi fruiting and seedling recruitment in a stand treated to reduce rat abundance and a neighbouring control stand. Fruit retention increased following treatment but seedling establishment did not. Although rat control improves the ability of hau kuahiwi to retain fruit, other, presently unknown inhibitors to seed dispersal, germination, and/or seedling development remain. Seed and seedling predation by other species, competition from numerous invasive plant species, unsuitable climate, and/or other factors may be primary inhibitors in the absence of rats, but we emphasize that progressive isolation of these factors at individual hau kuahiwi life stages may be necessary to identify the remaining threats to the conservation of this critically endangered plant.

## Introduction

Biological invasions of rodents and other vertebrate animals have been especially problematic in remote island ecosystems [1–7]. In instances where rodent removal has been successful, or where the effects of invasive rats have been compared to rat-free islands, the impacts on vegetation have been dramatic. For example, rats depressed tree and shrub recruitment in off-shore islands of New Zealand to the point of some species becoming locally rare [8]. Rat eradication efforts on Palmyra Atoll led to increased seedling recruitment of five native tree species, including two rare species [9]. Few other pre-removal plant surveys exist because researchers involved in rat removal planning have not been focused on impacts to plant communities.

Multiple invasive species or natural enemies may affect one or more life stages of a plant, sometimes with contrasting or non-linear effects [10]. Pollination, seed development, dispersal, and germination all must succeed in order for plants to propagate, but these processes are sensitive to introduced competitors and predators at multiple levels [11]. One difficulty in identifying the most-limiting factors to native plants in heavily-invaded ecosystems is that many invasive species have overlapping niche attributes, such as diet or behavior [12]. However, even if all limiting factors are identified at one life stage, subtle effects on other life stages of native plants may be present. For example, intense seed removal may mask the fact that high levels of competition exist for seedling establishment [13], especially in regards to rare plant species.

*Hibiscadelphus giffardianus* (hau kuahiwi in Hawaiian language) is one such rare plant. It is an endemic to the island of Hawai’i with invasion-induced threats to various stages of its life cycle. The *Hibiscadelphus* genus (Malvaceae) is a group of six endemic Hawaiian species, including three that are critically endangered and three that are extinct [14]. Hau kuahiwi have been reduced to a single tree on three separate occasions [15], including when it was first discovered in 1911 in Kīpuka Puaulu, Hawai’i Volcanoes National Park. Seeds were taken from this individual and cultivated in a garden [16] and today the species is only found in a single population of 223 planted trees at Kīpuka Puaulu and Kīpuka Kī, two small, neighboring stands in Hawai’i Volcanoes. Despite persistent conservation efforts, threats such as introductions of numerous non-native competitors and predators, the loss of natural pollinators, climate change, and more than a decade of inadvertent hybridization with a related species have left hau kuahiwi on the brink of extinction [15, 17]. Until this study, the foremost among these threats has been believed to be the consumption of fruit and seeds by *Rattus rattus* (black rats) [17], which consume hau kuahiwi bark, nectar, flowers, and fruit and as much as 90 percent of its seed crop [18], largely inhibiting the only existing population of the plant from reaching life stages beyond the production of fruit. Rats are known to severely affect hau kuahiwi fruit production, strip bark, and depredate buds and flowers to reach the nectar [18]. Trees surrounded by sheet metal flashing to exclude rats have been found to exhibit higher numbers of flowers and calyxes [17], which are the precursor stages to fruit development. While other species of rats (*R*. *exulans and R*. *norvegicus*) are present in the study area, their numbers are low relative to the abundance of *R*. *rattus* [19]. Mice (*Mus musculus*) also were present, but their abundance was not estimated. Spurr et al. [19] reported relatively low numbers of mice from the same area.

Natural recruitment of hau kuahiwi has been virtually nonexistent, with the natural establishment of one or more seedlings having been observed only three times in the wild up to the commencement of this study. After observing black rat predation of hau kuahiwi seeds, Pratt et al. [17] recommended the development of techniques to temporarily reduce rat populations during periods of peak fruit production, for example, through the use of approved toxicants.

This study evaluates the effect of rodent control (targeting *Rattus rattus*) on the ability of hau kuahiwi to complete its life cycle. Our objective was to determine whether reduced rat populations would yield increased fruit retention and seedling recruitment of hau kuahiwi (by fruit retention we mean fruit that remain attached to the branches of the tree). This aim was achieved in regards to fruit retention, but if there was any effect on recruitment, numbers were so sparse that we were not able to detect it.

### Study site

The study was conducted in two neighboring kīpuka, Kīpuka Puaulu and Kīpuka Kī, which are patches of well-developed forest surrounded by more recent lava flows (i.e. <750 years old) [20]. These kīpuka lie 2.5 km northwest of Kīlauea Caldera in Hawai`i Volcanoes National Park on the island of Hawai`i (19.441° N, 155.304° W). Kīpuka Puaulu spans approximately 100 ha, ranging from 1,200–1,280 m asl. Kīpuka Kī is approximately 90 ha at an elevation ranging from 1,210–1,350 m asl. The two kīpuka are separated by 800 m and thus have very similar climatic conditions. The climate is typically wet with mean annual precipitation of approximately 1,840 mm [21]. June–September are the driest months, with monthly rainfall <25 mm while wetter months may exceed 100 mm. Mean annual temperatures range from 13–17°C [22] with little seasonality. Soils in both sites are deep ash-derived silt-loam with >4,000 year-old substrate [20]. The 700 × 700 m rat-removal treatment area fell within Kīpuka Kī, straddling Mauna Loa Road and spanning an elevation range from 1210–1310 m asl, very similar to the elevation range of Kīpuka Puaulu. Hau kuahiwi in Kīpuka Puaulu served as a control, which was last reported to have higher numbers of hau kuahiwi fruit [17]. We monitored 181 mature, fruit-bearing individuals of this species, of which there exist 223 total. Ideally we would have included additional control and treatment sites but were constrained by the current range of hau kuahiwi, which does not extend beyond these two kīpuka. Logistical constraints limited rat removal to 49 ha (700 x 700 m). In order to establish a rodenticide application plot of this size and maintain a functional buffer zone between control and treatment sites, we conducted the treatment in one kīpuka and used the neighboring kīpuka as a control. The 42 hau kuahiwi not monitored in this study fell on the fringe or exterior of the treatment plot, and thus were not included in the analysis.

While the two kīpuka, which contain the entire distribution of hau kuahiwi, are in close proximity to one another and are similar in climate, soil properties, flora, fauna, and land use history, the number of fruit per tree in Kīpuka Kī (treatment) exceeded that of Kīpuka Puaulu (control) prior to the application of rodenticide. The inverse was true in 2007–2008, the time of the most recent survey preceding our study [17]. This discrepancy may be related to the fact that rats in Kīpuka Puaulu outnumbered rats in Kīpuka Kī at a rate of more than two to one at the time of our study, potentially leading to greater fruit retention prior to treatment in Kīpuka Kī. Thus, any effect of treatment on fruit retention or recruitment must necessarily be significantly different from both pre-treatment levels and any effect among control plots in order to be justified.

**Fig 1:**
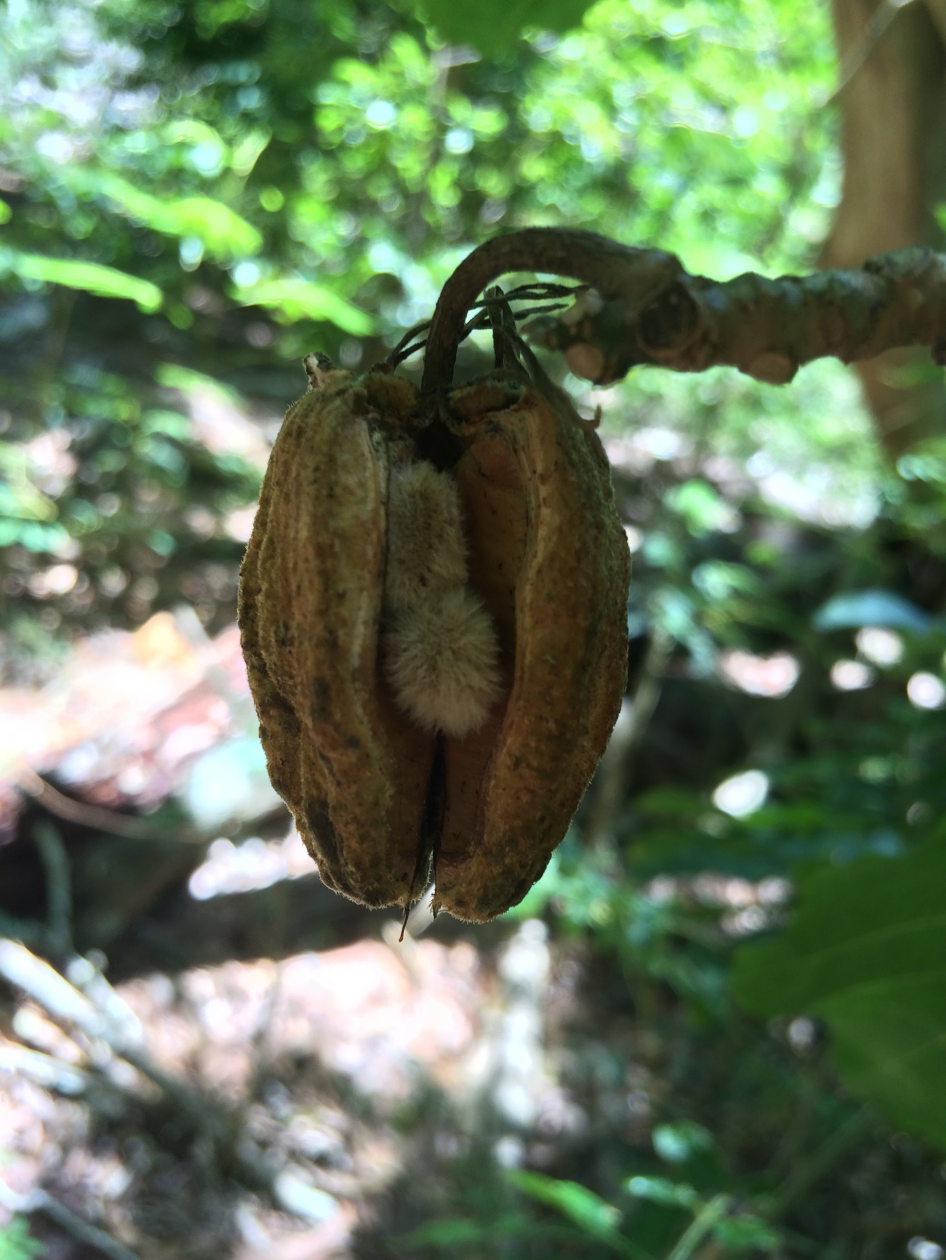
Desiccated hau kuahiwi (*Hibiscadelphus giffardianus*) fruit and exposed seeds.

Hau kuahiwi (S1, S2 and S3 Figs) flower and fruit continually throughout the year with slight peaks, but the seasonality of these peaks is not consistent among individuals or years [17]. The plant’s pollination syndrome is unknown, but is believed to be biotic through one or more species of native and non-native birds and insects [17]. A single tree may produce an average of 7–12 flowers at a time on average, but larger trees can produce more than 30 flowers at a time (fruit and flower productivity is related to number of branches and tree size) [17]. Peaks in flower production have been observed in winter and spring months, with flowering throughout the entire year [17]. Hau kuahiwi fruit are pink dehiscent capsules that open naturally via desiccation as they mature and, if not consumed, seeds fall naturally to the ground (Fig 1). No known dispersers presently exist and the seeds are understood to disperse across only short distances through gravity. A single hau kuahiwi fruit produces nine to fifteen seeds, and a single tree produces an average of 1.7 fruit capsules per year [17]. Greenhouse seed germination trials yielded a 23.2% germination rate out of 595 seeds after 16 trials spanning four years [17]. Prior to this study, only a very few seedlings (believed to be <10, exact number not known) of this species had ever been observed to establish naturally in the wild, thus, seedling survivorship rates are largely unknown.

## Materials and methods

The protocol used in this study was approved by the Institutional Animal Care and Use Committee of the University of Hawaii (Protocol Number 15–2038). All efforts were made to minimize suffering. This study was conducted under National Park Service Scientific Research and Collecting Permit HAVO-2017-SCI-0029.

### Experimental design

Each month from October 2016 through May 2017, the number of open fruit (seed-release stage) were recorded on individual hau kuahiwi trees in Kīpuka Puaulu (*n* = 71) and within the treatment area inside Kīpuka Kī (*n* = 110) for a total of 1448 observations (S1 Data). Trees included in the study were all planted between 1997–2001, with the exception of 3 trees still surviving in Kīpuka Puaulu that were planted between 1951–1964. All trees included in the dataset were sexually mature and known to have produced fruit during 2016–2017. Numbers of mature fruit were recorded each month; thus, fruit count data represent fruit retention of each tree.

Monthly surveys were performed on field plots extending 3 m beyond the circumference of each hau kuahiwi canopy in all directions (*n* = 181) and the presence of any newly established seedlings was recorded (S1 Data; National Park Service Scientific Research and Collecting Permit HAVO-2017-SCI-0029). All newly established seedlings were located and recorded early in their growth (height < 3 cm). Because of high mortality rates (>60%; S1 Data), after February we placed protective exclosures (1 cm mesh size, designed to exclude non-native Kalij pheasant (*Lophura leucomelanos*), which was identified as a potential but unconfirmed threat to hau kuahiwi) around remaining and new seedlings, as these are the only living individuals of this critically endangered plant to have established in the wild. Monthly hau kuahiwi surveys between the two sites were separated by no more than one day.

Rat population control was initiated in January 2017, immediately after hau kuahiwi surveys took place, and continued through the study. Rats were controlled in Kīpuka Kī using 0.45 kg of 0.005 percent diphacinone bait blocks, specifically Ramik Mini Bars (HACCO Inc., Randolph, Wisconsin) secured in tamper-proof Protecta bait stations (Bell Laboratories, Madison Wisconsin) designed to exclude non-target species. Stations were spaced every 50 m in the 700 × 700 m grid, and such applications typically begin to reduce rat populations within four to six days of bait distribution [19]. Baits were regularly replenished to 0.45 kg of bait blocks in order to maintain continuous accessibility to rats through August 2017. Initially, when bait consumption was high, bait stations were refilled twice per week, but the interval was extended to once per month as bait consumption declined. We removed dead rats and mice (and one mongoose [*Herpestes javanicus*]) that were occasionally found in or near bait stations.

To monitor rat abundance in both the treatment and non-treatment sites before and after the distribution of rodenticide, we conducted live mark-recapture surveys in 300 × 300 m plots in each study site (S2 Data). Rats were captured using 49 wire basket traps spaced 50 m apart and baited with coconut chunks, following the general procedures of Spurr et al. [19]. Rats were ear-tagged before release to prevent double-counting recaptured individuals, and the rate of mortality or escape from traps before ear-tagging was <10 percent at all sites. Rats were trapped during two sessions spaced ~1 month apart, each consisting of four consecutive nights in October–November 2016 (before the January application of rodenticide) and again in April–May 2017. We trapped for a total of eight nights per season to increase the probability of catching rats that might have been trap-shy, were inhibited by other rats, or encountered traps that were closed due to interference from mice and other animals. Kīpuka Kī was trapped for one additional night in November due to excessive mongoose interference with traps. We encountered only *R*. *rattus*, but the two other, less abundant rat species (*R*. *exulans* and *R*. *norvegicus*) incidental in the study area would also have been similarly affected by rodenticide treatment [19].

Additional surveys of hau kuahiwi were conducted in June and August 2017, more than seven months after rodenticide treatment began and more than five months after observed increases in open fruit (average seedling establishment occurs within one-to-three months of seed release, with a maximum of six months [17]).

We caution that the nature of the range and population of hau kuahiwi may somewhat limit the scope of our results. However, as there are presently no hau kuahiwi beyond the range of our study area, this limited scope is sufficient for our intentions. We acknowledge that site and treatment may be confounded. We have designed our experiment and analysis accordingly, comparing not only geographically separate treatment and control groups but also pre-and post-treatment [23–24]. We also consider these limitations when discussing results.

### Analysis

Black rat abundance was estimated as the number of individuals per 100 trap-nights with correction for trap failures or other causes of trap inaccessibility. Traps potentially unavailable to new (unmarked) rats due to incidental triggering, bait-robbing without capture, or occupancy by a recaptured (marked) rat or other species were given a value of 0.5 corrected trap-night (CTN) [25–26]. A chi-square test was used to evaluate differences in rat abundance in Fall 2016 and Spring 2017, before and after rodenticide application.

We tested whether rat treatment affected seedling recruitment and fruit production by running a two-sample t-test on the differences in mean response between the periods before and after rat treatment by kīpuka, following the methods of Newton et al. [27]. In other words, for each tree we calculated the mean response of the four observations before rat treatment, and subtracted from it the mean response of the four observations after rat treatment. We then performed a two-sample Welch’s t-test on these differences by kīpuka. Welch’s t-test is robust against samples having unequal variance and unequal sample sizes, as was the case in this study. This procedure was done for number of open fruit (seed-release stage) and number of seedlings. A census of all known hau kuahiwi trees within the plot boundaries that were known to produce fruit was included in the dataset.

## Results

Rat activity was significantly reduced in the treatment area (χ = 19.55, df = 1, *p* < 0.001), but not in the control (χ = 0.32, df = 1, *p* = 0.569) relative to pre-treatment levels. At Kīpuka Kī (treatment), the rat capture rate was 7.51 rats/100 CTN in Fall 2016 (before treatment) and 0.83 rats/100 CTN in Spring 2017 (after), which indicates a reduction of 89 percent in rat capture, which is presumably correlated with rat activity. At Kīpuka Puaulu (non-treatment), the capture rate was 17.93 rats/100 CTN in Fall of 2016 and 16.28 rats/100 CTN in Spring 2017. Although we did not estimate the abundance of mice before or after treatment with rodenticide, the dead mice that we occasionally found inside and outside bait stations indicated that the mouse population may have declined to some extent. However, mice have a smaller home range size than rats and thus would not be expected to exhibit a population decline of the same magnitude as we observed for rats, given the spatial arrangement of bait stations.

**Fig 2:**
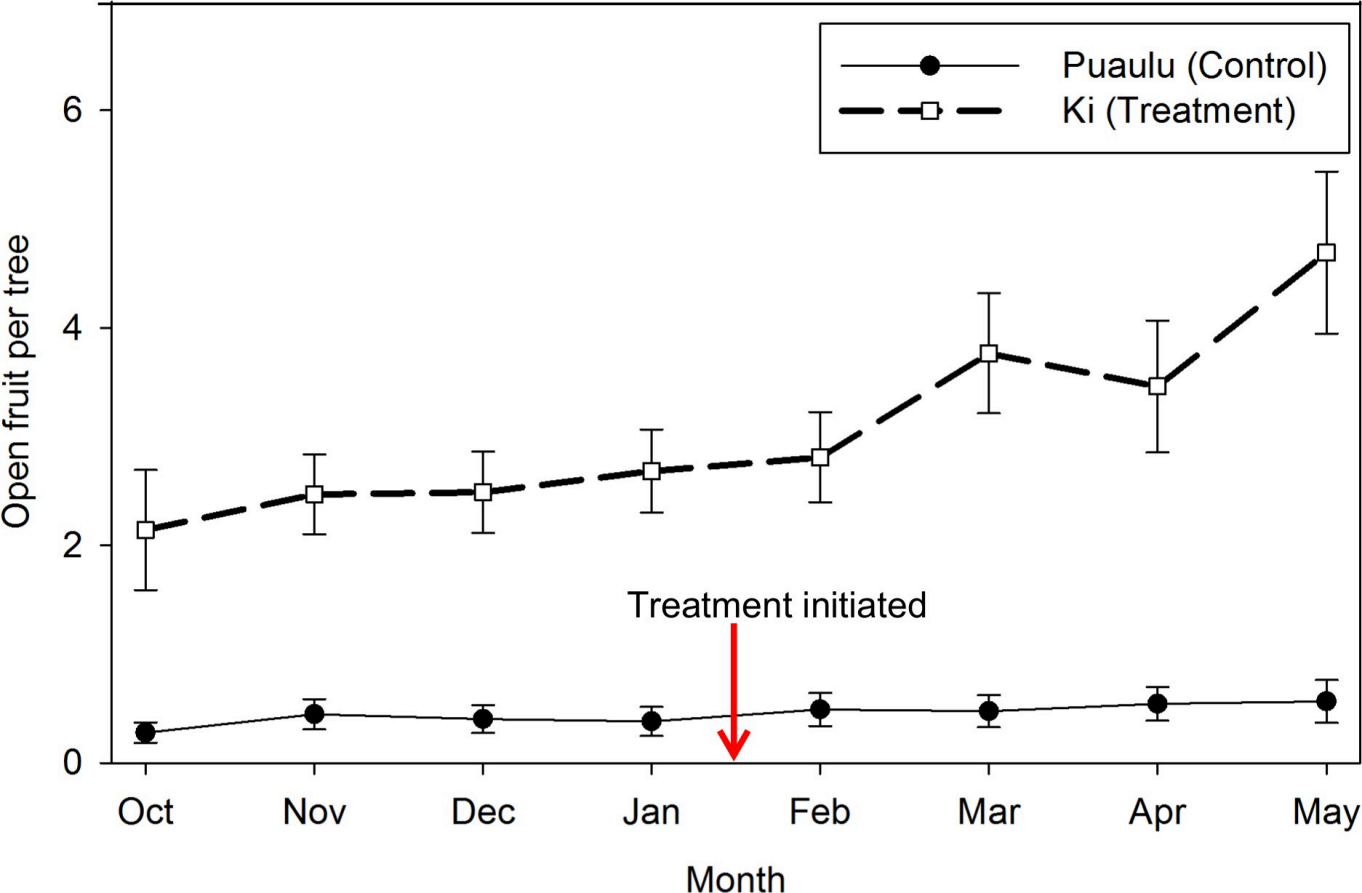
Hau kuahiwi (*Hibiscadelphus giffardianus*) open fruit retention. Fruit retention was observed monthly over four months before and four months during rat control (median ±SE). Rat control was initiated immediately after January data collection.

The number of open fruit in Kīpuka Kī (treatment) increased more than in Kīpuka Puaulu (control; Fig 2). The differences in mean number of open fruit (*t* = 4.035, df = 117.04, *p* < 0.001) between the periods before (Oct–Jan) and after (Feb–May) rat treatment were significantly more negative for the treatment site versus the control site (Fig 2). This equated to a mean difference of >2 fruit per tree, more than double the mean pre-treatment levels in the stand, and more than triple the *annual* average reported in previous years[17]. The differences in mean seedling recruitment between the periods before and after treatment were not significantly different for the two sites (*t* = -0.996, df = 81.578, *p* = 0.322;).

Recruitment levels remained extremely low: We recorded only eight seedlings throughout the study (three in Kīpuka Puaulu, five in Kīpuka Kī), given 181 mature trees over 12 months. Although we make no conclusive findings regarding seedling survivorship, the majority of new seedlings in both kīpuka were no longer present within one month of the first time we observed them (they may have been depredated, as no remnant of the plants remained). After placing exclosures around seedlings following February data collection, no additional seedlings were lost in the final six months of monitoring. Further research is needed to conclusively determine whether exclosures significantly increase survivorship.

## Discussion

Black rats have previously been identified as a primary limiting factor to hau kuahiwi fruit production and retention [17–18], and we observed evidence of rat activity on hau kuahiwi trees and on one occasion observed a rat in hau kuahiwi using night vision goggles. When black rat abundance was reduced by 89 percent over the course of seven months, we observed increased fruit retention but no increase in seedling establishment. Furthermore, the increased fruit retention came in the late spring, while previously reported peaks of hau kuahiwi fruit abundance have been observed in winter months [17]. The observed more-than-doubling of retained fruit that opened to release seeds is biologically significant given the critically endangered status of the species, consistently low fruit set in past reports [17], and fact that natural recruitment has only been observed three times in 110 years prior to this study. The significantly increased fruit retention but lack of natural recruitment reported in the present study indicate that although rats may inhibit fruit retention on a biologically meaningful level, one or more additional inhibitors to hau kuahiwi conservation exist at the seed dispersal, germination, and/or seedling establishment stages. These inhibitors to hau kuahiwi recruitment need to be further studied to ensure successful regeneration of this endangered species in the wild.

That the increase in fruit retention and seed release after rat removal in Kīpuka Kī was greater than natural seasonal variation in this or past studies [17] suggests that the pattern was due to more than simple within-year variation. In contrast to Kīpuka Kī, fruit abundance of the control site remained within the range reported in the 2006–2008 surveys. Together, these data suggest that the increase in fruit retention seen here was due to rat removal rather than other site differences. Our t-test results indicate differences in fruit retention between the treatment and control, but due to a pseudoreplicated study design [28] we cannot definitively assign the causality to rat removal *per se*. Furthermore, year-to-year variation was not directly addressed in this study. However, given that rats were removed from this site, that rats are predators of hau kuahiwi fruit and are detrimental to seed set [17–18], and that the two kipuka are the same flow age, have similar soil, climate, and land-use history, all lines of evidence lead us to believe that rat removal was the causal factor.

We observed that half of the already sparse natural recruitment (*n* = 8 new seedlings total throughout the study period) were removed within one to two months from seedling establishment at both sites, including some before and some after treatment. Albeit small, this number of recruiting hau kuahiwi is noteworthy given the virtual absence of natural seedling establishment of the species in its entire recorded history. We cautiously draw interpretations from these recruitment data as no other naturally established seedlings of the species presently exist. Survival rates of previously outplanted hau kuahiwi seedlings in the study area have been documented to exceed 70 percent; out of 15 hau kuahiwi successfully grown from sown seed in a previous study, nine persisted for >1 year, with no significant difference in survivorship between those growing inside versus outside rodent exclosures [17]. Thus, Pratt et al. [17] observed that rats feed on hau kuahiwi seed in the field, but found no evidence that they feed on seedlings, suggesting that limiting factors other than rats are important for recruitment success.

Possible inhibitors to recruitment remaining after rat control include seed and seedling predation by invasive species including birds (e.g., Kalij pheasant, which consume seedlings of other rare Hawaiian plants in Kīpuka Kī and Kīpuka Puaulu [17]), mice (which may have remained after rodenticide treatment), slugs [29], and a small number of remaining rats, as well as competition from invasive grasses, pathogens, and novel climatic conditions that are not suitable for germination [30]. In addition, the loss of natural pollinators, nectar robbing [31], inbreeding depression due to low genetic diversity, sporophytic self-incompatibility, and insufficient seed production remain as possible threats [17], though we believe these are unlikely to be most-limiting factors as they would be manifest at earlier life stages (e.g. failure to produce fruit and seed). Further research is required to confirm the identity of additional inhibiting factors. Potential next steps include exclosure studies combined with camera traps [32] and experiments where seeds and seedlings are placed/planted and tracked. This study suggests that the removal of a single inhibiting factor can reveal the presence of additional inhibitors to which rare plants are not adapted. The stepwise removal of known impeding factors may serve to identify presently unknown threats to rare plant conservation and inform management efforts to bolster populations into the future.

## Supporting information

S1 Data Hau kuahiwi fruit retention and seedling recruitment. Monthly data on fruit retention and recruitment of hau kuahiwi (*Hibiscadelphus giffardianus*) in Kīpuka Puaulu and Kīpuka Kī from October 2016 through May 2017 (fruit) and August 2017 (recruitment) are contained in a Microsoft Excel file titled S1 Data.
(XLSX)

S2 Data Rat capture. A summary of rat captures during Fall 2016 and Spring 2017 trapping periods is provided in a Microsoft Excel file titled S2 Data.
(XLSX)

S1 Fig Hau kuahiwi (*Hibiscadelphus giffardianus*) flower and calyx. (TIF)

S2 Fig Hau kuahiwi (*Hibiscadelphus giffardianus*) with damaged petals and calyx, as is often observed after rat activity. (TIF)

S3 Fig Immature hau kuahiwi (*Hibiscadelphus giffardianus*) fruit. (TIF)
