# Supplementary figures and images for: Invasive rat control is an efficient, yet insufficient, method for recovery of the critically endangered Hawaiian plant hau kuahiwi (Hibiscadelphus giffardianus)
Source: PLoS One. 2018 Nov 28;13(11):e0208106. doi: 10.1371/journal.pone.0208106 (PMC6261625; doi:10.1371/journal.pone.0208106)

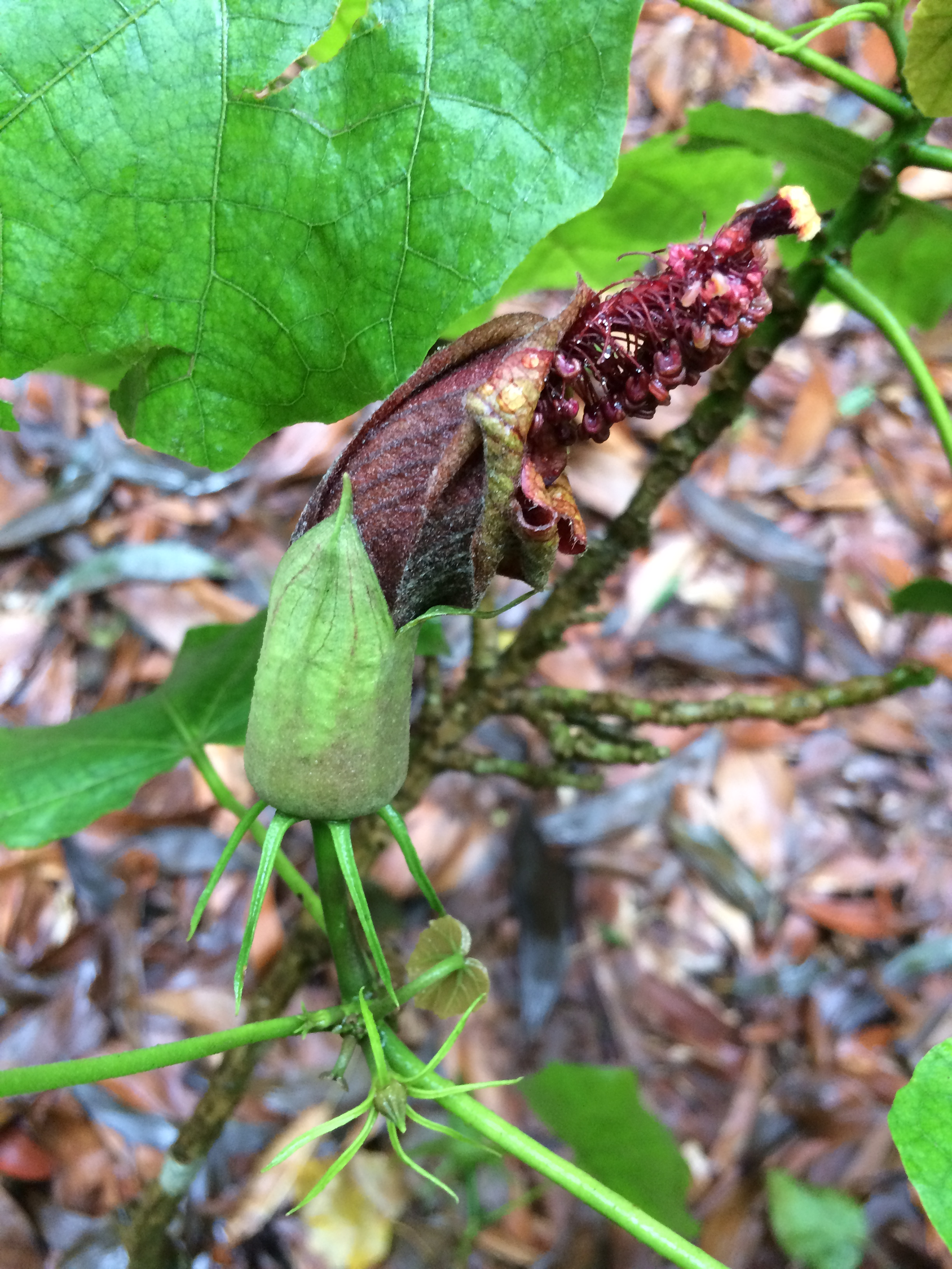

Supplement: S1 Fig — (TIF) [file pone.0208106.s003.tif]

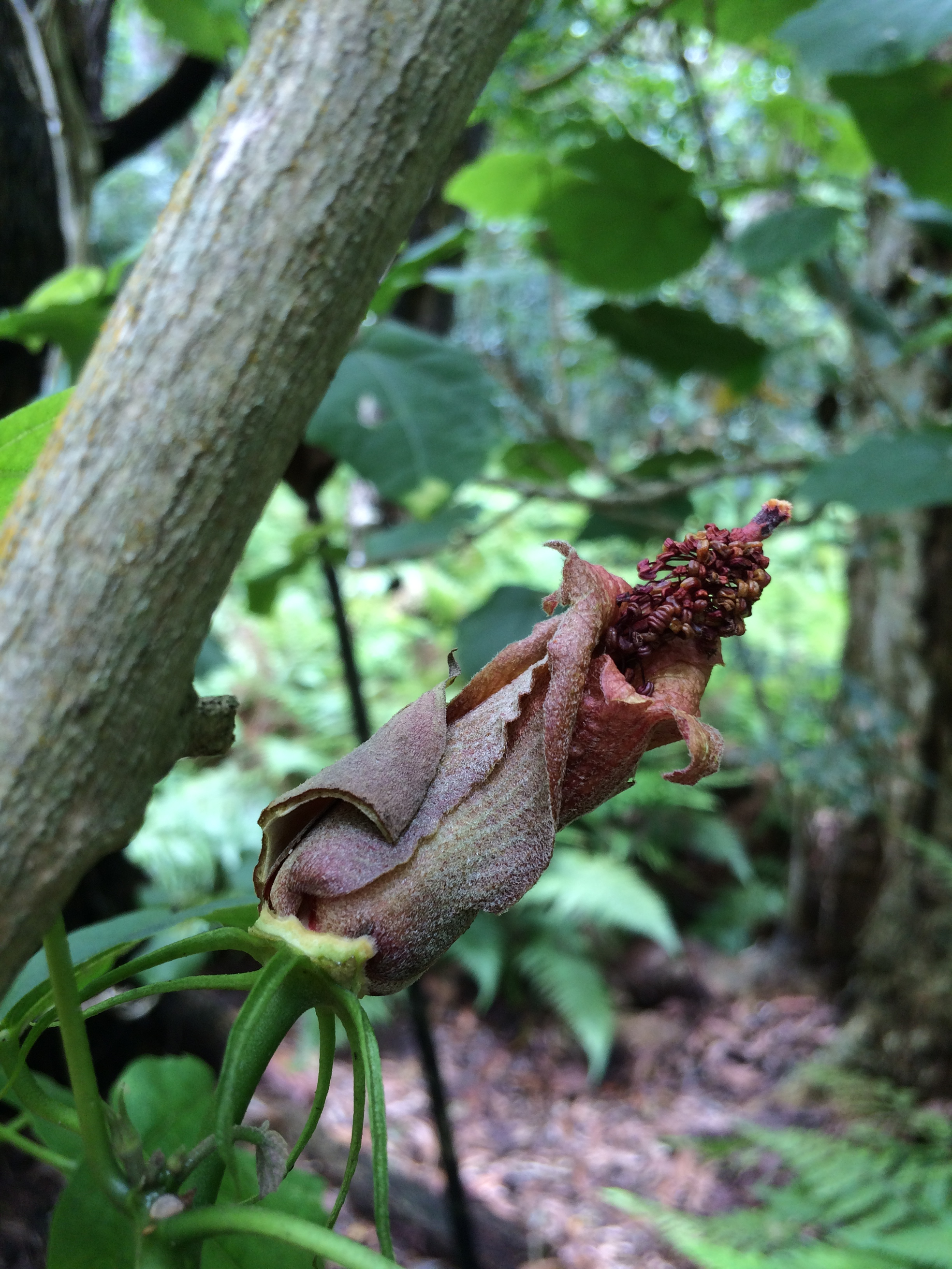

Supplement: S2 Fig — (TIF) [file pone.0208106.s004.tif]

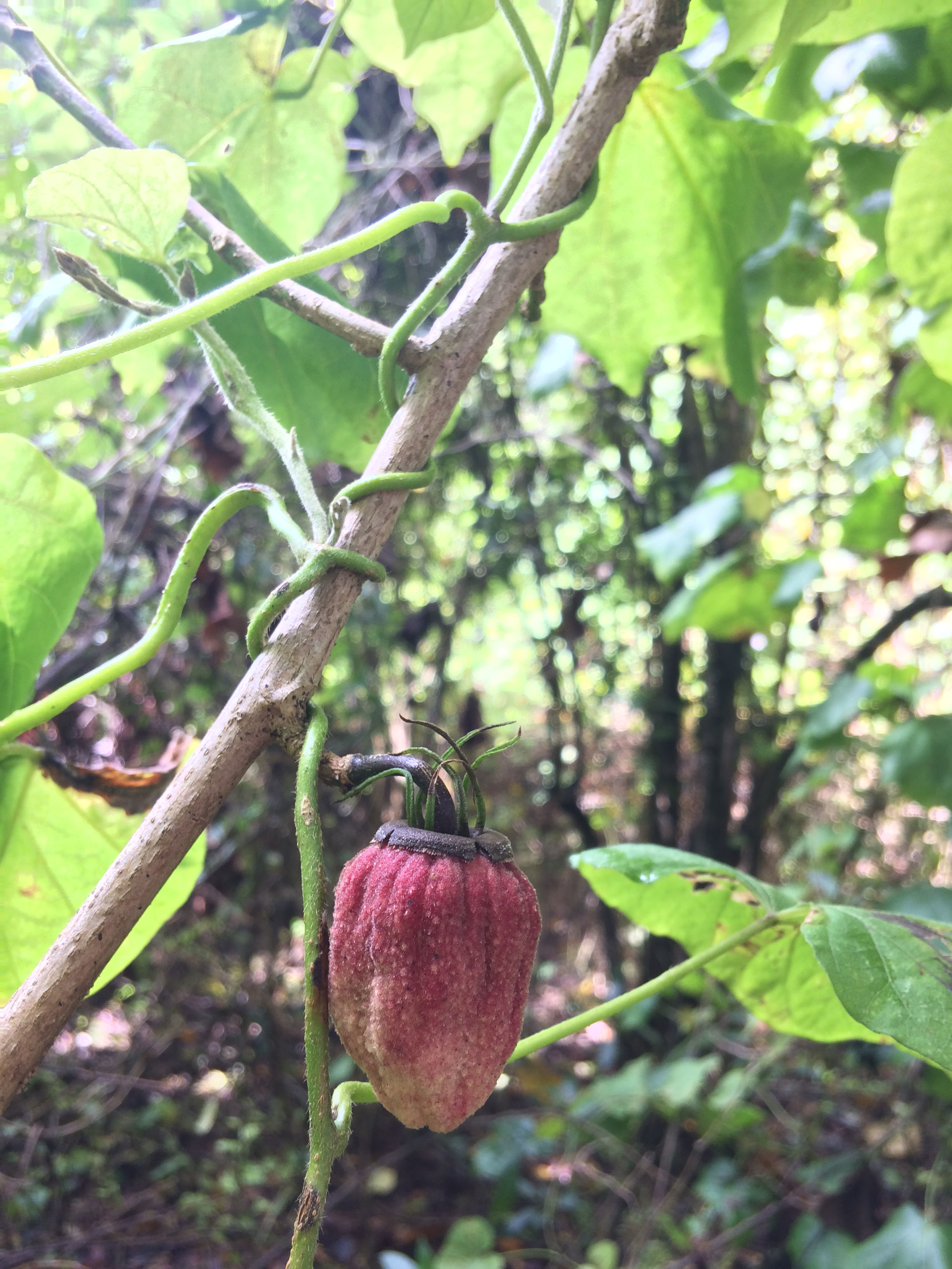

Supplement: S3 Fig — (TIF) [file pone.0208106.s005.tif]
